# High progesterone levels on the day after HCG injection has no effect on clinical pregnancy outcomes in *in vitro* fertilization-embryo transfer

**DOI:** 10.3389/fendo.2024.1372753

**Published:** 2024-04-16

**Authors:** Zhuo Liang, Qiuyan Huang, Jiwei Huang, Jinxiang Wu, Dingyuan Zeng, Pinxiu Huang

**Affiliations:** ^1^Center of Reproductive Medicine, Guangzhou Women and Children’s Medical Center-Liuzhou Hospital, Liuzhou, Guangxi, China; ^2^Guangxi Clinical Research Center for Obstetrics and Gynecology, Liuzhou, Guangxi, China; ^3^Obstetrics and Gynecology Institute, Guangzhou Women and Children’s Medical Center-Liuzhou Hospital, Liuzhou, Guangxi, China; ^4^Center of Reproductive Medicine, Liuzhou Maternal and Child Health Hospital, Liuzhou, Guangxi, China; ^5^Graduate School, Guilin Medical College, Guilin, Guangxi, China; ^6^Department of Obstetrics and Gynecology, The First Clinical Medical College of Jinan University, Guangzhou, Guangdong, China; ^7^Affiliated Hospital of Youjiang Medical University for Nationalities, Baise, Guangxi, China; ^8^Department of Reproductive Medicine, the Second Affiliated Hospital of Fujian Medical University, Quanzhou, Fujian, China

**Keywords:** progesterone, endometrial receptivity, *in vitro* fertilization-embryo transfer, clinical pregnancy outcome, human chorionic gonadotropin injection progesterone

## Abstract

**Background:**

This study investigates the potential impact of high progesterone (P) level on the day following human chorionic gonadotropin (HCG) injection on the clinical pregnancy outcomes of *in vitro* fertilization-embryo transfer (IVF-ET).

**Methods:**

Retrospective analysis was conducted on 6418 cycles of IVF-ET performed at Liuzhou Maternal and Child Health Hospital between August 2020 to December 2021. Excluding cycles with progesterone levels ≥1.5ng/ml on HCG injection, a total of 781 cycles were identified according to the standard, and they were divided into five groups according to the progesterone level on the day after HCG: Group A: progesterone level < 2.5 ng/ml (n = 128); Group B: 2.5 ng/ml ≤ progesterone level < 3.5 ng/ml (n = 174); Group C: 3.5 ng/ml ≤ progesterone level < 4.5 ng/ml (n = 153); Group D: 4.5 ng/ml ≤ progesterone level < 5.5 ng/ml (n = 132); Group E progesterone level ≥5.5 ng/ml(n=194). Comparative analyses of clinical data, including general clinical data, and clinical pregnancy outcomes such as clinical pregnancy rate, miscarriage rate, and live birth rate were performed among these groups.

**Results:**

There were significant differences in estradiol levels on HCG injection, but there were no differences in available embryo rate, clinical pregnancy rate, miscarriage rate, and live birth rate. Binary logistic regression analysis showed that there was no significant correlation between P level on the day after HCG injection and the live birth rate.

**Conclusion:**

Under the condition of low P level on HCG injection, high progesterone levels on the day after HCG injection does not affect the clinical pregnancy outcomes of IVF-ET.

## 1 Introduction

In the realm of assisted reproductive technologies, *in vitro* fertilization-embryo transfer (IVF-ET) stands as the ultimate resource for infertile women in their quest for pregnancy. The realization of an optimal clinical pregnancy outcome is influenced by many factors, including age, ovulation induction scheme, thickness of the transplanted endometrium, quality and quantity of transplanted embryos, endometrial receptivity, and so on. At present, many studies have proved that progesterone level on human chorionic gonadotropin (HCG) injection reaching or exceeding 1.5ng/ml will significantly affect the clinical pregnancy outcome in IVF-ET. The primary mechanistic underpinning this impact lies in the increased progesterone levels disrupting endometrium receptivity, leading to premature transformation of endometrium, and inducing asynchronous development between the embryo and the endometrium (Bosch et al., 2010) (1).

The theory of action mechanism was proposed earlier by Forman RG, emphasizing the excessive binding of progesterone receptors on the endometrium, thereby expediting endometrium transformation (Forman et al., 1989) (2). Labarta and Shen’s research further suggested that high progesterone level on HCG injection would lead to changes in gene expression profiles in the endometrium, resulting in premature endometrium transformation and decreased receptivity (Labarta et al., 2011; Shen et al.,2018) (3, 4). However, whether progesterone levels on the day after HCG injection exhibit a similar mechanistic influence as high progesterone level on HCG - prompting early endometrium transformation to the secretory phase and, consequently, affecting endometrial receptivity or further affecting the clinical pregnancy outcome of IVF-ET through other mechanisms - is a subject meriting thorough examination (Bosch et al., 2003; Liu et al., 2017; Xiong et al., 2017; De et al., 2020; Zhang et al., 2021) (5–9). In order to eliminate the influence of high progesterone level on HCG injection, this retrospective study mainly analyzes the impact of progesterone levels on the day after HCG injection on the clinical pregnancy outcome of IVF-ET when the progesterone level on HCG injection is less than 1.5ng/ml, so as to offer valuable insights that can guide clinical practice and serve as a reference for practitioners in the field.

## 2 Object and method

### 2.1 Research object

This study adopts a retrospective analysis approach, focusing on 6,418 IVF-ET cycles conducted at Liuzhou Maternal and Child Health Hospital, Guangxi from August 2020 to December 2021. The inclusion criteria were as follows: (1) The ovulation induction scheme is gonadotropin releasing hormone (GnRH) agonist long scheme; (2) Fresh embryo transfer was conducted with at least one high-quality embryo transferred. Cleavage stage embryos are classified according to Istanbul standard (Alpha Scientists in Reproductive Medicine and ESHRE Special Interest Group of Embryology, 1989) (10). Classification is based on cell number, morphology and debris content. High-quality embryos (grade I and II) means that the number of cells exceeds 6 on the third day, the cell morphology is uniform and the fragment content is less than 20%. Blastocyst stage embryos are classified according to Gardner standard (Gardner et al., 2000) (11). Classification is based on the expansion ratio, the development of inner cell mass and the appearance of outer trophoblast cells. High-quality embryos means that the score of 3BB and above (AA/AB/BA/BB); (3) The thickness of endometrium on the day of transplantation should be at least 7 mm; (4) Double embryo transfer at the cleavage stage or single embryo transfer at the blastocyst stage; (5) The patients included in the study once. Exclusion criteria encompassed: (1) Progesterone ≥1.5ng/ml on HCG injection; (2) Presence of pathological changes affecting transplantation, such as reproductive tract infections, uterine cavity effusion, hysteromyoma, adenomyosis, endometrial polyps, endometriosis, hydrosalpinx, etc.; (3) Anatomical abnormality of the reproductive tract; (4) Missing visit or missing data; (5) Cancellation of transplantation for various reasons; (6) Recurrent spontaneous abortion and recurrent implantation failure patients. After inclusion and exclusion, a total of 781 IVF-ET cycles were included in the study and divided into five groups according to the progesterone level on the day after HCG injection: Group A: progesterone < 2.5 ng/ml (n = 128); Group B: 2.5 ng/ml ≤ progesterone < 3.5 ng/ml (n = 174); Group C: 3.5 ng/ml ≤ progesterone < 4.5 ng/ml (n = 153); Group D: 4.5 ng/ml ≤ progesterone < 5.5 ng/ml (n = 132); Group E progesterone ≥5.5 ng/ml(n=194). The study was approved by the ethics committee of Guangzhou Women and Children’s Medical Center-Liuzhou Hospital, China.

### 2.2 Methods

(1) Ovulation-promoting and fertilization modes: The ovulation induction strategy employed the GnRH agonist long scheme. When the diameter of the two dominant follicles exceeded 18mm, HCG was injected intramuscularly. After 36 hours, the eggs were retrieved transvaginally under ultrasound, and conventional corpus luteum support was given after the eggs were taken. Embryos transfer occurred 3-5 days later, the cleavage stage embryos or blastocyt stage embryos transfer rates similar between the groups.
(2) Measurement index collection method: HCG stimulation was implemented at nine o’clock on HCG injection, blood samples used for progesterone measurement was collected at nine o’clock on the day after HCG injection.
(3) Observation indicators: Clinical pregnancy rate, miscarriage rate and live birth rate. Ultrasound examination was performed on the 28th day after transplantation, confirming intrauterine gestational sac as indicative of clinical pregnancy. Abortion before the 12th week of pregnancy is defined as miscarriage. Live birth is defined as a fetus reaching 28 weeks and surviving. Calculation formulas are as follows: Clinical pregnancy rate= (clinical pregnancy cycles ÷ total transplantation cycles) × 100%; Miscarriage rate= miscarriage cycles ÷ clinical pregnancy cycles × 100%; Live birth rate= live birth cycles ÷ total transplantation cycles × 100%.
(4) Statistical analysis: SPSS 25.0 software (IBM, New York, USA) was used for statistical analysis. The measurement data were expressed by mean ± standard deviation (X ± S). Analysis of continuous variables was conducted using nonparametric tests for independent samples, and analysis of classified variables was conducted using the Chi-square test. Binary logistic regression analysis was used to analyze the correlation between progesterone levels on the day after HCG injection and live birth rate. Results were presented as odds ratios (OR) with corresponding 95% confidence intervals (CIs). P-values less than 0.05 were considered statistically significant.

## 3 Results

### 3.1 Research object

**Figure 1:**
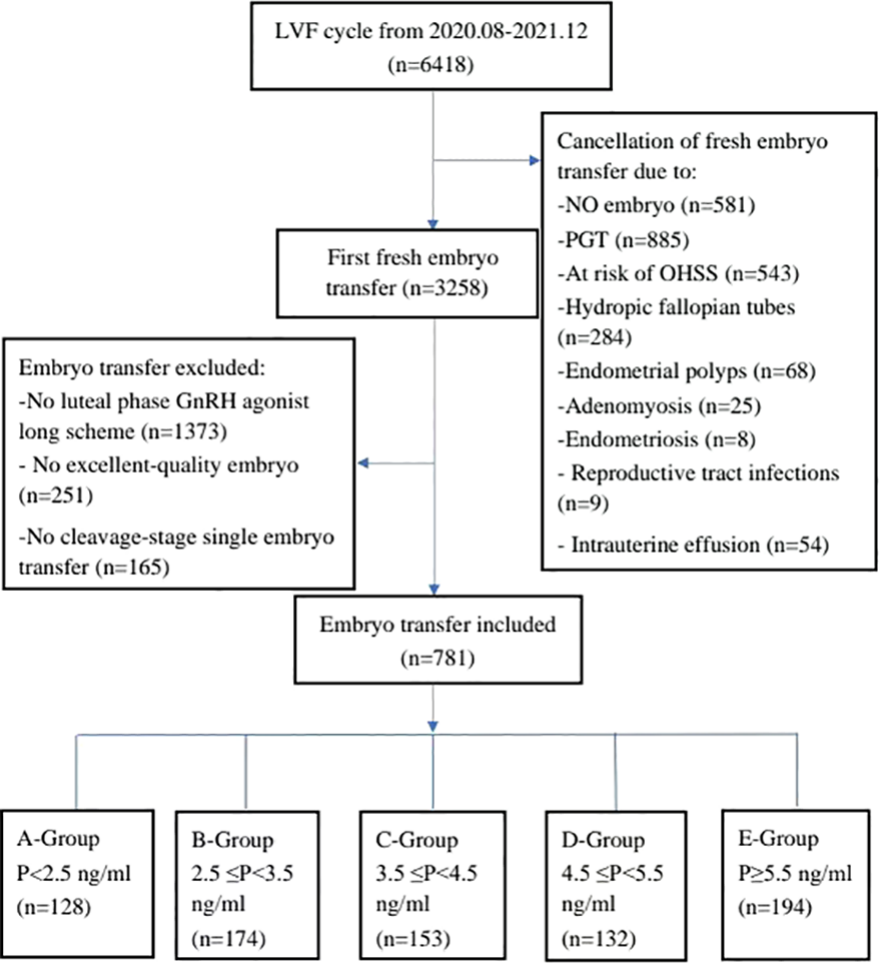
Flow chart of the inclusion and exclusion criteria.

Initially, a total of 6,418 cycles were considered for inclusion in the study. However, 3,160 cycles were cancelled due to various reasons, including no embryo transfer (n = 581); Preimplantation genetic testing for aneuploidy (n=885); Intrauterine effusion (n = 54); Ovarian irritation sign (n = 543); Hydrosalpinx (n = 284); Genital tract infection (n = 9); Thin endometrium on the day of transplantation (n = 214); Endometrial polyps (n = 68); Endometriosis (n = 8); Adenomyosis (n = 25); and other reasons (n=489). Of the remaining 3,258 cycles, 2,477 cycles were excluded according to the standard, including missing data (n = 728); No GnRH agonist long scheme (n = 1,373); High quality embryos were not transplanted (n = 251); and Single embryo transfer at cleavage stage (n=165). Consequently, a final cohort of 781 cycles was included in the analysis, and they were divided into five groups based on progesterone levels on the day after HCG injection: Group A: P < 2.5 ng/ml (n = 128); Group B: 2.5 ng/ml ≤ progesterone < 3.5 ng/ml (n = 174); Group C: 3.5 ng/ml ≤ progesterone < 4.5 ng/ml (n = 153); Group D: 4.5 ng/ml ≤ progesterone < 5.5 ng/ml (n = 132); Group E progesterone ≥5.5 ng/ml(n=194) (**Figure 1**).

### 3.2 General clinical data

There were significant differences in basal follicular stimulating hormone (FSH) and infertility factor (unexplained infertility and other factors) among groups, but no statistically significant differences in age, infertility duration, infertility types, basal Luteinizing hormone (LH), basal estradiol (E2) and infertility factors (tubal factor, male factor and ovulation failure) (**Table 1**).

**Table 1:** General Clinical Data for Each Group.

	A-group P<2.5 (n=128)	B-group 2.5≤P<3.5 (n=174)	C-group 3.5≤P<4.5 (n=153)	D-group 4.5≤P<5.5 (n=132)	E-group P≥5.5 (n=194)	P value
Female age (years)	33.99 ± 4.90	33.32 ± 4.23	32.83 ± 4.50	33.63 ± 4.45	33.43 ± 4.11	0.206
Infertility duration (years)	4.95 ± 3.89	4.74 ± 3.74	4.67 ± 3.61	4.82 ± 3.46	5.46 ± 4.10	0.302
Infertility type (primary/secondary)	45/83	55/119	52/101	45/87	72/122	0.864
Antral follicle count	10.91 ± 5.78	11.76 ± 4.96	11.29 ± 4.26	11.72 ± 4.33	11.93 ± 4.32	0.073
Basal FSH (IU/L)	5.94 ± 2.12	5.47 ± 1.61	5.31 ± 1.25	5.30 ± 1.21	5.13 ± 1.18	0.006
Basal LH (IU/L)	3.27 ± 1.65	3.41 ± 1.57	3.45 ± 2.07	3.40 ± 1.45	3.44 ± 2.09	0.646
Basal E2 (pg/mL)	46.93 ± 77.25	42.02 ± 38.42	42.13 ± 43.74	40.87 ± 46.50	36.46 ± 20.04	0.804
Infertility factor (tubal factor)	81/128	110/174	109/153	94/132	145/194	0.080
Infertility factor (male factor)	18/128	33/174	32/153	21/132	30/194	0.506
Infertility factor (unexplained infertility and other factors)	19/128	22/174	6/153	12/132	13/194	0.008
Infertility factor (ovulation failure)	10/128	9/174	6/153	5/132	6/194	0.343

### 3.3 Clinical pregnancy outcome

While significant differences were identified in estradiol upon HCG injection, there were no differences in transplantation day endometrium, available embryo rate, clinical pregnancy rate, miscarriage rate, or live birth rate (**Table 2**).

**Table 2:** Clinical Outcomes for Each Group.

	A-group P<2.5 (n=128)	B-group 2.5≤P<3.5 (n=174)	C-group 3.5≤P<4.5 (n=153)	D-group 4.5≤P<5.5 (n=132)	E-group P≥5.5 (n=194)	P value
E2 level on HCG injection (pg/mL)	1096.66 ± 595.33	1559.97 ± 619.93	1918.72 ± 684.93	2178.87 ± 753.04	2588.14 ± 841.84	<0.00001
Transplantation day endometrium (mm)	12.19 ± 2.50	12.08 ± 2.37	12.58 ± 2.61	12.11 ± 2.44	12.18 ± 2.49	0.497
Available embryo rate (%)	554/590 (93.90%)	995/1074 (92.64%)	985/1076 (91.54%)	957/1033 (92.64%)	1630/1787 (91.21%)	0.209
Clinical pregnancy rate (%)	69/128 (53.91%)	102/174 (58.62%)	83/153 (54.25%)	75/132 (56.82%)	109/194 (56.19%)	0.923
Clinical miscarriage rate (%)	11/69 (15.94%)	13/102 (12.75%)	9/87 (10.34%)	14/75 (18.67%)	12/109 (11.01%)	0.440
Live birth rate (%)	57/128 (44.53%)	86/174 (49.43%)	70/153 (45.75%)	60/132 (45.45%)	91/194 (46.91%)	0.925

### 3.4 Progesterone level on the day after HCG of live birth and no live birth

Analysis revealed no significant difference in progesterone level on the day after HCG injection between live birth and no live birth (**Table 3**).

**Table 3:** Progesterone level on the day after HCG between live birth and no live birth.

	Live birth (n=364)	No live birth (n=417)	P value
P level on the day after HCG injection	4.40 ± 2.12	4.33 ± 1.94	0.945

### 3.5 Binary logistic regression analysis

The OR with the corresponding 95% CI and P values for each parameter was included in the regression model. The only significant P value (<0.0001) was female age (OR=0.905, 95% CI: [0.872; 0.939]). After adjusting for female age, infertility duration, whether primary infertility, basal FSH level, basal LH level, basal E2 level, E2 level on HCG injection, and transplantation day endometrium, the increase in progesterone levels on the day after HCG injection has no effect on the live birth rate (**Table 4**).

**Table 4:** Binary Regression Model.

	Significance	EXP(B)	The 95% confidence interval for the EXP (B)	
			Lower limit	Upper limit
Female age	.000	.908	.874	.943
Infertility duration	.351	.981	.942	1.022
Whether primary infertility	.971	1.006	.731	1.384
Antral follicle count	.588	1.009	.976	1.044
Basal FSH level	.119	.918	.824	1.022
Basal LH level	.771	1.013	.929	1.104
Basal E2 level	.864	1.000	.997	1.004
E2 level on HCG injection	.915	1.000	1.000	1.000
Transplantation day endometrium	.215	1.038	.978	1.101
P level on the day after HCG injection				
P<2.5	Reference			
2.5≤P<3.5	.911	1.033	.590	1.807
3.5≤P<4.5	.614	1.129	.704	1.812
4.5≤P<5.5	.618	.889	.561	1.411
P≥5.5	.876	.964	.606	1.533

## 4 Discussion

IVF-ET remains a focal point of assisted reproduction, and the challenge of optimizing success rate prompts continued investigation into multifaceted factors that influence clinical pregnancy outcomes. Among these factors, endocrine hormones, such as progesterone and estradiol, play crucial roles in modulating endometrial transformation and receptivity, ultimately impacting the success of clinical pregnancy outcome. Numerous studies have demonstrated the adverse effects of high progesterone level on HCG injection on clinical pregnancy outcome in IVF-ET (Lou et al., 2018; Cai et al., 2013; Kolibianakis et al., 2012) (12–14). However, there are still few studies the correlation between high progesterone levels on the day after HCG injection and clinical pregnancy outcomes. Our investigation leaded to the conclusion that a high progesterone level on the day after HCG injection has no effect on the clinical pregnancy outcome of IVF-ET, particularly when the progesterone level on HCG injection is low.

In this study, we analyzed the correlation between progesterone level on the day after HCG injection and the clinical pregnancy outcome of IVF-ET. Patients were divided into groups according to the progesterone level on the day after HCG injection, and there were no significant differences in clinical pregnancy rate, miscarriage rate and live birth rate among the groups. Subsequent binary regression analysis validated these findings, confirming the absence of a significant correlation between the progesterone level on the day after HCG injection and live birth rate. This led us to infer that variations in the progesterone level on the day after HCG injection do not yield significant changes in endometrial receptivity.

Contrastingly, Zhu’s study divided patients into groups based on the ratio of progesterone levels on HCG injection and the day after HCG injection, demonstrating that an increase in progesterone levels had no effect on implantation rate and clinical pregnancy rate (Zhu et al., 2014) (15). However, the lack of a clear correlation between progesterone levels on HCG injection and those on the day after HCG injection raises uncertainties regarding the reliability of determining the influence of progesterone levels on the clinical pregnancy outcome based on this ratio.

In Shen’s study, 941 transplantation cycles were analyzed and divided into three groups according to the progesterone level on the day after HCG injection. Notably, there were no significant differences in the implantation rate, clinical pregnancy rate, miscarriage rate, and live birth rate among these groups (Shen et al.,2018) (4). However, the implantation rate and clinical pregnancy rate in the group with the highest progesterone level on the day after HCG injection showed a downward trend when compared with the other two groups. Shen’s study supports the overarching conclusion that the progesterone level on the day after HCG injection has no effect on clinical pregnancy rate, miscarriage rate, and live birth rate. Nevertheless, it is crucial to acknowledge that Shen’s study did not control for the potential influence of progesterone level on HCG injection. The group with the highest progesterone level on the day after HCG injection also exhibited higher progesterone levels on HCG injection than the other two groups. Their conclusion that high progesterone level on the day after HCG injection’s implantation rate and clinical pregnancy rate showed a downward trend needs further validation and scrutiny.

Some divergent perspectives have been presented in other studies compared to our findings. Burns’s study revealed that even after controlling the progesterone level on HCG injection, a high progesterone level on the day after HCG injection will significantly reduce the clinical pregnancy rate (Burns et al., 1994) (16). However, this study has limitations, including a smaller dataset and technical constraints that impacted endometrial transformation within 24 hours after egg retrieval. This limitation greatly affected the progesterone level on the day after HCG injection and resulted in suboptimal endometrial receptivity. Check’s study, on the other hand, reported both excessively low and high progesterone levels on the day after HCG injection could lead to unfavorable clinical pregnancy outcomes (Check et al., 2009) (17). However, this study did not control for the progesterone level on HCG injection. Consequently, the reference significance of the conclusions drawn from both studies is constrained.

In the broader context, there is a scarcity of known studies exploring the influence of the progesterone level on the day after HCG injection on clinical pregnancy outcomes. Moreover, most existing studies have not controlled for the potential influence of progesterone levels on HCG injection. In our study, progesterone on HCG injection was controlled to be less than 1.5 ng/ml, in order to eliminate the risk of premature transformation of endometrium to the secretory phase. In the IVF-ET cycle, the day after HCG injection is equivalent to the day before ovulation in the natural cycle, marking the normal initiation of progesterone to rise at this time. This rise is crucial for the transformation of the endometrium from the proliferative phase to the secretory phase, facilitating entry into the endometrial receptive phase. This transformative process requires a certain time for progesterone to exert its effects. Therefore, in the case of low progesterone levels on HCG injection, a subsequent elevation in progesterone levels on the day after HCG injection does not necessarily indicate an advanced state of endometrial transformation. According to the results of this study, we found that when the progesterone level is low on HCG injection, a high progesterone level on the day after HCG injection will not impact the clinical pregnancy outcome of IVF-ET.

At the same time, this study is retrospective, introducing potential unmeasurable confounding factors. Therefore, caution is advised when considering the guidance for clinical decision-making based on a high progesterone level on the day after HCG injection in IVF-ET. Further research will refine methodologies and larger sample sizes is warranted to corroborate and extend our findings.

## Data availability statement

The raw data supporting the conclusions of this article will be made available by the authors, without undue reservation. The datasets for this study can be found at dx.doi.org/10.6084/m9.figshare.25019792.

## Author contributions

ZL: Methodology, Validation, Writing – original draft, Writing – review & editing. QH: Writing – original draft, Writing – review & editing. JH: Data curation, Methodology, Writing – review & editing. JW: Data curation, Writing – review & editing. DZ: Data curation, Methodology, Writing – review & editing. PH: Data curation, Methodology, Writing – original draft, Writing – review & editing, Funding acquisition.
