# Supplementary material for: High progesterone levels on the day after HCG injection has no effect on clinical pregnancy outcomes in in vitro fertilization-embryo transfer
Source: Front Endocrinol (Lausanne). 2024 Apr 16;15:1372753. doi: 10.3389/fendo.2024.1372753 (PMC11059087; doi:10.3389/fendo.2024.1372753)
Supplement: Supplementary file 1 [file Table_1.docx]

As for single embryo transfer at the cleavage stage, a total of 103F-ET cycles were included in the study and divided into five groups according to the progesterone level on the day after HCG injection: Group A: progesterone < 2.5 ng/ml (n = 26); Group B: 2.5 ng/ml ≤ progesterone < 3.5 ng/ml (n = 30); Group C: 3.5 ng/ml ≤ progesterone < 4.5 ng/ml (n = 21); Group D: progesterone ≥4.5 ng/ml(n=26).

There were no statistically significant differences in age, infertility duration, infertility types, basal Luteinizing hormone (LH), follicular stimulating hormone (FSH), basal estradiol (E2) and infertility factors (Table SI).

While significant differences were identified in estradiol upon HCG injection, there were no differences in transplantation day endometrium, available embryo rate, clinical pregnancy rate, miscarriage rate, or live birth rate (Table SII).

Analysis revealed no significant difference in progesterone level on the day after HCG injection between live birth and no live birth (Table SIII).

The OR with the corresponding 95% CI and P values for each parameter was included in the regression model. The significant P value (<0.0001) was female age (OR=0.726, 95% CI: [0.609; 0.867]) and transplantation day endometrium (OR=1.314, 95% CI: [1.009; 1.710]). After adjusting for female age, infertility duration, whether primary infertility, basal FSH level, basal LH level, basal E2 level, E2 level on HCG injection, and transplantation day endometrium, the increase in progesterone levels on the day after HCG injection has no effect on the live birth rate (Table SIV).

| Supplementary Table SI General Clinical Data for Each Group | | | | | |
| --- | --- | --- | --- | --- | --- |
|  | A-group  P<2.5 (n=26) | B-group  2.5≤P<3.5 (n=30) | C-group  3.5≤P<4.5 (n=21) | D-group  P≥4.5 (n=26) | P value |
| Female age (years) | 35.08v4.22 | 34.43±3.89 | 34.24±4.30 | 33.12±4.14 | 0.247 |
| Infertility duration (years) | 4.85±4.06 | 5.00±3.42 | 5.19±4.25 | 4.88±2.94 | 0.892 |
| Infertility type (primary/secondary) | 6/20 | 6/24 | 5/16 | 6/20 | 0.987 |
| Antral follicle count | 9.27±5.63 | 10.80±3.45 | 9.71±3.55 | 11.23±4.32 | 0.026 |
| Basal FSH (IU/L) | 5.90±1.39 | 5.54±1.53 | 5.49±1.99 | 4.86±1.29 | 0.073 |
| Basal LH (IU/L) | 3.14±2.04 | 2.74±0.97 | 3.32±1.85 | 3.63±2.02 | 0.356 |
| Basal E2 (pg/mL) | 53.99±68.34 | 46.08±26.49 | 46.36±36.56 | 42.20±26.34 | 0.851 |
| Infertility factor (tubal factor) | 17/26 | 22/30 | 16/21 | 20/26 | 0.784 |
| Infertility factor (male factor) | 2/26 | 4/30 | 1/21 | 4/26 | 0.606 |
| Infertility factor (unexplained infertility and other factors) | 6/26 | 2/30 | 3/21 | 1/26 | 0.127 |
| Infertility factor (ovulation failure) | 1/26 | 2/30 | 1/21 | 1/26 | 0.955 |

| Supplementary Table SII Clinical Outcomes for Each Group | | | | | |
| --- | --- | --- | --- | --- | --- |
|  | A-group  P<2.5 (n=27) | B-group  2.5≤P<3.5 (n=30) | C-group  3.5≤P<4.5 (n=21) | E-group  P≥4.5 (n=26) | P value |
| E2 level on HCG injection (pg/mL) | 740.04±403.55 | 1436.94±553.47 | 1760.09±850.37 | 2356.59±759.20 | <0.00001 |
| Transplantation day endometrium (mm） | 11.60±2.61 | 11.49±1.63 | 11.58±2.47 | 11.65±2.25 | 0.993 |
| Available embryo rate (%) | 69/73 | 119/142 | 72/88 | 158/184 | 0.103 |
| Clinical pregnancy rate (%) | 11/26 | 9/30 | 7/21 | 9/26 | 0.809 |
| Clinical miscarriage rate (%) | 3/11 | 2/9 | 2/7 | 1/9 | 0.809 |
| Live birth rate (%) | 6/26 | 7/30 | 5/21 | 8/26 | 0.813 |

| Supplementary Table SIII Progesterone level on the day after HCG between live birth and no live birth | | | |
| --- | --- | --- | --- |
|  | Live birth (n=26) | No live birth (n=77 | P value |
| P level on the day after HCG injection | 3.80±1.76 | 3.57±1.64 | 0.561 |

| Supplementary Table SIV Binary Regression Model | | | | |
| --- | --- | --- | --- | --- |
|  | Significance | EXP(B) | The 95% confidence interval for the EXP (B) | |
|  |  |  | Lower limit | Upper limit |
| Female age | .000 | .726 | .609 | .867 |
| Infertility duration | .697 | .966 | .810 | 1.151 |
| Whether primary infertility | .078 | 4.018 | .856 | 18.846 |
| Antral follicle count | .169 | .889 | .752 | 1.051 |
| Basal FSH level | .171 | .702 | .423 | 1.165 |
| Basal LH level | .251 | .789 | .526 | 1.183 |
| Basal E2 level | .164 | 1.011 | .996 | 1.026 |
| E2 level on HCG injection | .304 | 1.000 | 1.000 | 1.001 |
| Transplantation day endometrium | .043 | 1.314 | 1.009 | 1.710 |
| P level on the day after HCG injection |  | | | |
| P<2.5 | Reference | | | |
| 2.5≤P<3.5 | .515 | 2.146 | .215 | 21.409 |
| 3.5≤P<4.5 | .418 | 2.040 | .363 | 11.459 |
| P≥4.5 | .549 | 1.704 | .298 | 9.758 |
